# The Alternative Pre-hospital Pathway team: reducing conveyances to the emergency department through patient centered Community Emergency Medicine

**DOI:** 10.1186/s12873-021-00536-x

**Published:** 2021-11-18

**Authors:** Andrew Patton, Cathal O’Donnell, Owen Keane, Kieran Henry, Donal Crowley, Adrian Collins, Eoghan Redmond, Nicky Glynn, Martin Dunne, Conor Deasy

**Affiliations:** 1 Emergency Department, Cork University Hospital, Wilton, Cork, Ireland; 2 Medical Directorate, National Ambulance Service, Health Service Executive, Limerick, Ireland; 3 National Ambulance Service, Health Service Executive, Cork, Ireland; 4 School of Medicine, University College Cork, Cork, Ireland

**Keywords:** Community emergency medicine, Non-conveyance, Pre-hospital, Ambulance, Alternative pathways, Low acuity, Paramedicine

## Abstract

**Background:**

Internationally increasing demand for emergency care is driving innovation within emergency services. The Alternative Pre-Hospital Pathway (APP) Team is one such Community Emergency Medicine (CEM) initiative developed in Cork, Ireland to target low acuity emergency calls.

In this paper the inception of the APP Team is described, and an observational descriptive analysis of the APP Team’s service data presented for the first 12 months of operation. The aim of this study is to describe and analyse the APP team service.

**Methods:**

The APP Team, consisting of a Specialist Registrar (SpR) in Emergency Medicine (EM) and an Emergency Medical Technician (EMT) based in Cork, covers a mixed urban and rural population of approximately 300,000 people located within a 40-min drive time of Cork University Hospital. The team are dispatched to low acuity 112/999 calls, aiming to provide definitive care or referring patients to the appropriate community or specialist service.

A retrospective analysis was performed of the team’s first 12 months of operation using the prospectively maintained service database.

**Results:**

Two thousand and one patients were attended to with a 67.8% non-conveyance rate. The median age was 62 years, with 33.0% of patients aged over 75 years. For patients over 75 years, the non-conveyance rate was 62.0%. The average number of patients treated per shift was 7. Medical complaints (319), falls (194), drug and alcohol related presentations (193), urological (131), and respiratory complaints (119) were the most common presentations.

**Conclusion:**

Increased demand for emergency care and an aging population is necessitating a re-design of traditional models of emergency care delivery. We describe the Alternative Pre-Hospital Pathway service, delivered by an EMT and an Emergency Medicine SpR responding to low acuity calls. This service achieved a 68% non-conveyance rate; our data demonstrates that a community emergency medicine outreach team in collaboration with the National Ambulance Service offering Alternative Pre-Hospital Pathways is an effective model for reducing conveyances to hospital.

**Supplementary Information:**

The online version contains supplementary material available at 10.1186/s12873-021-00536-x.

## Background

Demand for Emergency Care internationally is increasing year on year. In Ireland, there were 1.4 million emergency department (ED) presentations in 2019, a 2.6% increase on 2018, [1] and a 19% increase from 1.18 million 5 years prior in 2014 [2]. Similarly, demand for emergency ambulances has increased dramatically, with the National Ambulance Service (NAS) responding to 348,053 emergency calls in 2019, [1] a 19% increase from 293,095 calls in 2014 [2]. This increased demand has been reflected in the international literature [3, 4].

Internationally, Emergency Medical Services (EMS) have seen growing use of ambulances for non-emergency calls with many patients who access these services not requiring emergency interventions by pre-hospital practitioners [5, 6]. Currently in Ireland, Pre-Hospital Emergency Care Council (PHECC) clinical practice guidelines (CPGs) are built around conveyance to hospital unless the patient declines treatment [7]. Recognising that there is unsustainable increasing demand, EMS systems are changing their model of care from treat and transport, to acute mobile healthcare services where care is delivered at scene in an integrated model by accessing alternative pathways [8]. As part of the long-term evolution of the NAS, it has been moving from an Emergency Medical Service to a Mobile Medical Service (MMS) to ensure that patients receive the appropriate care in the most appropriate setting. The introduction of a telemedical clinical hub, a mental health signposting desk, Community Paramedics and other hospital avoidance models are examples of such developments [9].

The direction of healthcare policy in Ireland is changing. In May 2017, the Sláintecare Report was produced, [10] a cross-political party consensus document on a long-term policy direction for Ireland’s healthcare system. The report outlined an integrated model of care with the majority of healthcare being provided in the community to meet the needs of our older population with its more complex set of clinical and social care needs.

In line with the Sláintecare aims, [10] the Alternative Pre-Hospital Pathway (APP) Team was founded in November 2019, as a Community Emergency Medicine collaboration between Cork University Hospital (CUH) and the National Ambulance Service (NAS). The team tends to patients in the community, responding to low acuity 112/999 emergency calls with a view to intervening at the very start of the patient journey to provide definitive care to these patients in the community, or to refer them to the most appropriate community or specialist service. The aim of the initiative is to reduce conveyances to the ED, free up emergency ambulance resources to be available for critically ill or injured patients and provide excellent holistic patient care in the community.

The aim of this study is to describe and analyse the APP team service for the first 12 months of operation.

## Methods

### Pre-hospital model of care

The Pre-Hospital Emergency Care Council (PHECC) is the statutory regulator. Clinical levels currently in Ireland include Emergency Medical Technician (EMT), Paramedic and Advanced Paramedic (AP), with the recent proposed introduction of Specialist Paramedic to encompass Critical Care and Community Paramedics.

Pre-hospital emergency care in Ireland is provided by the National Ambulance Service (NAS) and the Dublin Fire Brigade (in the Dublin Metropolitan Region). Care is primarily paramedic led, with the exception of a few doctors around the country providing Pre-Hospital Emergency Medicine and critical care support on a voluntary basis. This is in contrast to EMS systems in continental Europe which rely heavily on physicians or specialist nurses for the provision of pre-hospital emergency care [11, 12].

The primary model of pre-hospital care provided in Ireland is that the majority of 112/999 calls result in an emergency ambulance being dispatched and the patient transported to an ED which is in contrast to the UK where a variety of responses are available including telephone advice, on scene treatment and discharge, and transport to ED or alternative services [8, 13]. A proportion of calls in Ireland are redirected to alternative pathways by the telemedical clinical hub.

### The APP team

The APP Team operates out of Cork University Hospital (CUH). The region is served by two EDs, CUH and the Mercy University Hospital (MUH) and three urgent care centres (UCC) which manage minor trauma. CUH is the tertiary referral centre for the south of Ireland serving an adult and paediatric population of over 1.1 million. In 2019 the CUH ED had 69,982 presentations [14]. The APP Team covers a population of approximately 300,000 people located within a 40 min drive time of CUH [15].

The APP Team started as a Monday to Friday, 12 pm–8 pm service, but with the surge of COVID-19 this was extended and is now a 7-day service operating 10 am-8 pm.The team consists of a Specialist Registrar (SpR) in Emergency Medicine from CUH ED and a NAS Emergency Medical Technician (EMT) in a NAS response vehicle. A SpR in Emergency Medicine is a doctor, who is at least 4 years post-qualification from university, having completed 3 years of Core Speciality Training in Emergency Medicine and is on the four-year training programme to become a Consultant in Emergency Medicine.

### Governance

The APP Team operate under a shared governance model; as an extension of the ED, clinical decisions and treatment provided by the SpR fall under the governance of CUH ED. Governance is shared with NAS, in particular relating to the tasking of the resource, the provision of a response vehicle and the performance of NAS staff working on the team.

Real-time clinical oversight for the team is provided by a Consultant in Emergency Medicine (EM) through telemedicine. Cases are reviewed on a daily basis by a Consultant in EM, and monthly clinical team meetings facilitate detailed discussions regarding cases and operational issues.

The two EDs operating in the region, CUH and MUH, are governed by the same EM Consultant group and share a common patient information system. This ensures visibility of any patients treated by the APP Team who were subsequently brought to either ED.

### Training

A structured orientation is provided to all team members prior to working on the APP Team including supernumerary operational shifts with an experienced team member. Attendance at monthly clinical team meetings and involvement in case discussions allow new members gain an understanding of care provided and commonly used alternative pathways.

### Equipment

A wide range of equipment (Table 1) and supplies are carried to facilitate provision of definitive care or initial treatment for a range of illnesses and injuries likely to be encountered. The vehicle is also stocked with advanced life support (ALS) equipment, a customised ALS response kit and a critical care drugs bag in the event of the resource being required at a higher acuity call for reasons of geography or resource availability.

**Table 1:** Equipment List

**Equipment** • Laptop & WiFi Dongle with remote access to Hospital Information System (Labs, Radiology, Discharge & OPD Letters) • iSTAT - Point of Care Blood Testing – Biochemistry, Troponin, Blood Gas Analysis • Point of Care Ultrasound • Lifepak 15 Manual Defibrillator / Monitor - 12 Lead ECG, etCo2, Pacing. • Suction Unit • Auriscope • Ophthalmoscope • SpO2 Monitor • Glucometer • Thermometer	**CUH Pre-Hospital Drugs Bag** • Sedation & Emergency Anaesthesia • Antiemetics • Critical Care / Resuscitation Drugs • Anti-Histamines • Antibiotics • Analgesics - IV, PO, PR, IM • Antiplatelets Agents • Nitrates • Steroids • Anticonvulsants • Anti-hypoglycaemics • Bronchodilators • Tranexamic Acid • Entonox / Methoxyflurane • Oxygen • IV Fluids	**APP Wound Management Bag** • Dressing Packs • Disposable Suture Sets • Sutures - Range of Sizes • Chlorhexidine, • Wound Stapler • Skin Glue • Range of Dressings & Sutures • Burn Gel & Dressings *Soft Tissue Injuries* • Wrist Splints • Shoulder Immobilisers *ENT* • Rapid Rhinos *Ophthalmology* • Morgan Lens Maternity Pack
**CUH Advanced Life Support Bag**	**APP Oral Medications Box**	**APP Urinary Catheter Bag**
• Advanced Airway Management - endotracheal tubes, bougies, iGel, front of neck access, etCO2 monitoring • Oxygen administration - non-rebreather masks, nasal prongs, nebulisers, C-Circuit, BVM • Thoracostomy & Chest Drain Equipment • IV Cannulation & IO Access Equipment • Haemorrhage Control - tactical dressings, combat tourniquets, Pelvic Binder, • Wound Stapler & Suturing Equipment	• Oral Analgesia - Paracetamol, Codeine, NSAIDs • Methoxyflurane • Anti-platelet Agents • GTN • Eye drops - Fluorescein, Tetracaine, Fusidic Acid • Bronchodilation - Inhalers & Nebules Oral Antibiotics • Antihistamines • Steroids • Antiemetics • Antispasmodics	• Urinary Catheters - range of sizes • Sterile Catheter Packs & Gloves • Instillagel • Chlorhexidine • Sterile Water • Bladder-Tip Syringe • Urine Dipsticks • Leg Bag & Night Bag • Urology Referral Forms • Patient Advice Leaflets
Personal Protective Equipment High Viz, Helmet COVID-19 Precautions - FFP2 & 3 Masks, Disposable Gowns,		**Paperwork** • Patient Care Report (PCR) • Discharge Letters • HSE Headed Paper • Prescription Pad • Ambulance PCR Advice Leaflets - Head Injury, COVID-19,

### Follow-up services

Prior to launch, we engaged with a wide range of stakeholders (Table 2) to assist us in developing alternative patient care pathways, and we continue to engage with additional services as the opportunities arise.

**Table 2:** APP Team Stakeholders

Clinical Operations Group, Emergency Department Geriatric Emergency Medicine Service Frailty Intervention Therapies Team CUH GP liaison CUH Flow Coordinator CUH Community Intervention Team Clinical Decision Unit ED CUH Syncope clinic Falls clinic Day hospital (Care of the Elderly patients) Acute Medical Unit staff at CUH, Mallow & Mercy Liaison Psychiatry & Alcohol Liaison Services staff CUH Medical Social Work CUH & MUH Social Inclusion Nurse MUH Specialist hospital teams Patient’s GP Community Pharmacy Drugs and Alcohol Community Service Arbor House Drug Treatment Centre Penny Dinners, St. Vincent’s Hostel and Cork Simon Hostel	

### Tasking

The National Emergency Operations Centre (NEOC) is a single centralised control centre; 112/999 calls are prioritised using Advanced Medical Priority Dispatch (AMPDS). The regional dispatcher screens the emergency calls and tasks the team to low acuity cases where they feel definitive care or an alternative pathway could be provided. (Table 3) Calls are also generated from paramedics and advanced paramedics (AP) at the scene who have assessed the patient and believe they would benefit from treatment by the APP Team (Crew Request). The team carries a mobile phone to discuss cases and potential treatment options with crews prior to formal tasking through NEOC.

**Table 3:** APP Team Dispatch Criteria

**Calls received from 112/999 Service:** Low acuity calls (Categorised as Alpha, Bravo & Omega by AMPDS) Minor Illnesses Minor Injuries & Wounds Urinary Catheter Issues Seizures Low Velocity Road Traffic Collisions (RTCs) Minor Falls (Patient Mobilising) Frequent Callers Frailty & Geriatric Syndromes Social Issues & Inclusion Health COVID-19 Remote Monitoring Clinical Reviews Paramedic / Advanced Paramedic Request from Scene (Crew Request) Other	

### Records

Details of each patient contact are recorded contemporaneously throughout the shift; patients are also registered on the hospital’s Integrated Patient Management System (iPMS). Independently, as with all 112/999 calls, NEOC maintains records of every incident including details of the emergency call and time stamps of allocated resources.

### Call categorisation

Calls were retrospectively categorised into presentations by the authors based on the clinician’s description of the case in the service database (Additional File 1).

### Data collection and analysis

For this retrospective observational review of activity, data was extracted from the APP Team’s service database. All patients seen by the APP Team in the 12-month period (3rd December 2019 - 3rd December 2020) were included. Database entries were excluded from analysis if the APP Team was stood down prior to reaching the patient or if consultation was provided exclusively by telemedicine. Formalised documentation of the ‘Source of Dispatch’ commenced 7 months into the project. Efforts were made to retrospectively record the source of dispatch from the clinician’s description of the case, but in many cases this was not possible.

After data collection, median and interquartile range were used for analysing the data with asymmetric distribution, categorical variables were described as frequencies and percentages.

Data analysis was performed in Microsoft Excel for Mac (Version 16.43). Ethics approval was granted by the Clinical Research Ethics Committee (CREC) of the Cork Teaching Hospitals based in University College Cork, and operational support was given by the National Ambulance Service Research Committee.

This report has been prepared in line with the Strengthening the Reporting of Observational Studies in Epidemiology (STROBE) statement [16].

### Patient and public involvement

Patients or the public were not involved in the design, conduct, or reporting or this study.

## Results

**Fig. 1:**
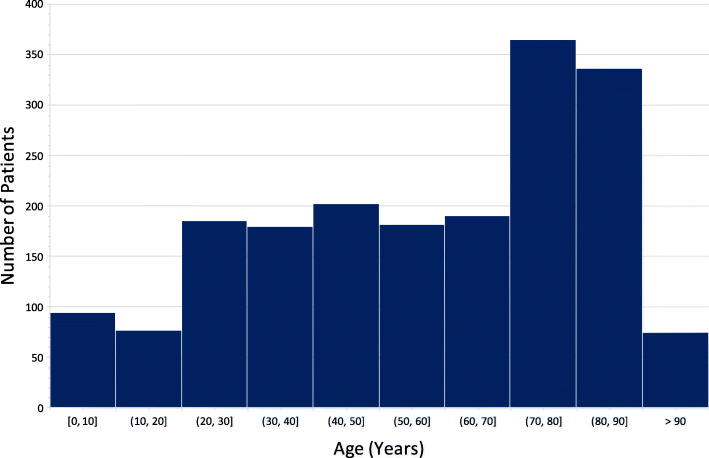
Age Distribution of Patients Treated by the APP Team

In the first year of operation (December 2019–December 2020), 2001 patients were seen by the APP Team over 297 shifts, averaging 7 patients per shift (Table 4). The median age was 62 years (IQR 38–79), ranging from 2 days old - 104 years (Fig. 1), with a balanced gender distribution, 53.7% male. We identified 62.5% (1250) of patients suitable for non-conveyance, 35.9% (718) were transported to hospital, and 1.6% [17] were pronounced dead on scene. In 170 cases (8.5%) the APP team was requested to attend as an Advanced Life Support (ALS) resource. Excluding these cases, the remaining sample of 1831 patients had a 67.8% non-conveyance rate. In our patient cohort, 33.0% of patients were aged over 75. Excluding 28 ALS calls, 62.0% of this subgroup were suitable for non-conveyance.

**Table 4:** Description of Patients

**Description**	**All Patients** *n*=2,001	**Conveyance** *n*= 718 (35.9%)	**Non-conveyance** *n*= 1250 (62.5%)	**Died on Scene** *n*= 33 (1.6%)
**Age (years)**	n=1889 (94.4%) documented			
Median (Interquartile Range)	62 (38-79)	66 (42-80)	59 (35-78)	65.5 (50-77)
>75 years n (%)	623 (33.0%)	246 (39.4%)	369 (59.2%)	8 (1.3%)
16-74 years n (%)	1147 (60.7%)	406 (35.3%)	721 (62.9%)	20 (1.7%)
<16 year n (%)	119 (6.3%)	33 (27.7%)	86 (72.3%)	0 (0.0%)
**Gender**	*n*=1786 (89.3%) documented			
Male n (%)	959 (53.7%)	344 (35.9%)	596 (62.1%)	19 (2.0%)
Female n (%)	827 (46.3%)	290 (35.1%)	529 (63.9%)	8 (1.0%)
**Source of Dispatch**	*n*=732 (36.6%) documented			
Primary Dispatch from NEOC n (%)	325 (44.4%)	136 (41.8%)	189 (58.2%)	
Crew Request n (%)	316 (43.2%)	103 (32.6%)	213 (67.4%)	
ALS Dispatch from NEOC n (%)	83 (11.3%)	65 (78%)	4 (4.8%)	14 (16.9%)
Other n (%)	8 (1.1%)	0	8 (100%)	
**Top 5 Presentations** n (%)	**Most encountered** Medical 319 (15.9%) Fall 194 (9.7%) Drug & Alcohol Related 193 (9.6%) Urology 131 (6.5%) Respiratory 119 (5.9%)	**Most commonly conveyed** Injuries: Major Trauma 10 (90.9%) Neurological 17 (89.5%) Injuries: Fractures 8 (80%) Surgical 36 (75%) Obstetrics & Gynae 13 (56.5%)	**Most commonly discharged** Palliative 14 (100%) Social 8 (88.9%) Injuries: Other 15 (88.2%) COVID-19 Related 54 (85.7%) Musculoskeletal 92 (83.6%)	

Neurological: New focal neurological deficit.

Surgical: Abdominal Pain, Vascular Presentations, Orthopaedic Issues (excluding acute injuries)

COVID-19 Related: COVID-19 Positive, COVID-19 Suspected, COVID-19 Swabs

Social: Medically fit patients suffering homelessness, elderly home help issues, poor social circumstances

Injuries: Other: Soft tissue injuries

### Non-conveyed

Of the 1250 patients suitable for non-conveyance, 877 (70%) patients had definitive care provided on scene and were discharged, and 373 (30%) patients had follow-up arranged prior to discharge, most commonly with their own GP (199 patients). Other alternative pathways accessed include: Outpatient Clinics, Clinical Nurse Specialists (e.g. Epilepsy and Diabetes), St Finbarr’s Assessment and Treatment Centre (ATC) for the elderly, Psychiatry Community Assessment Hub, a home visit by the Integrated Care Team or Public Health Nurse and scheduled appointments in the Acute Medical Unit.

### Conveyed

Of the 718 patients transported to hospital, 137 (19.1%) had an alternative pathway arranged by the APP Team. Direct admission by a speciality team (67 patients) or the ED Clinical Decision Unit (22 patients) were the most common alternative pathways used for those conveyed; as the patient had already been seen by an Emergency Doctor on the APP Team, they were able to bypass the ED process, and be seen directly by the specialty team. This helped reduce workload for the ED staff and expedited the patient journey through ED. Other common alternative pathways involved releasing emergency ambulances, through organising transport of patients by family members, taxi or scheduled non-urgent transfers by intermediate care ambulances (ICVs).

### Call types

**Fig. 2:**
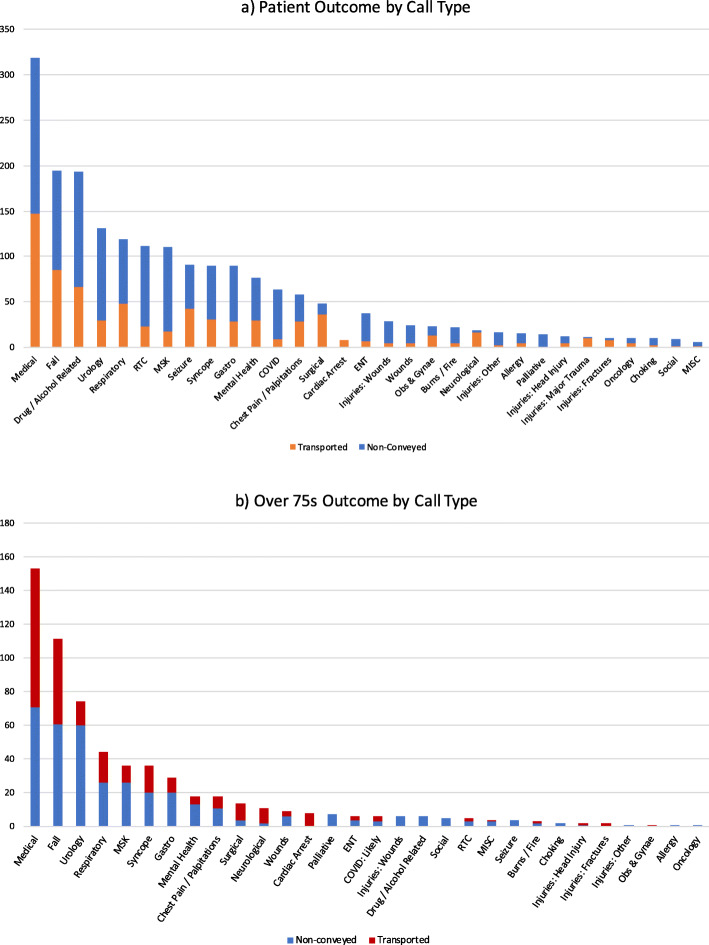
Patient Outcome by Call Type

The most common presentations (Fig. 2) were general medical (319), falls (excluding falls from significant height) (194), drug and alcohol related presentations (193), urology (including catheter care) (131) and respiratory complaints (excluding COVID-19 presentations) (119). Other notable presentations included minor road traffic collisions (112 patients) and musculoskeletal issues (110 patients) with 78.6% and 83.6% discharge rates respectively. General medical presentations included diabetic emergencies, confusion, lethargy, general decline, headaches, feeling generally unwell, dizziness, decreased mobility, poor oral intake and delirium.

### COVID-19

The period of data analysis encompasses Wave One and Wave Two of the COVID-19 Pandemic in Ireland. There were 63 (3.1%) patients treated by the APP Team who had COVID-19 related presentations, 86% of whom were suitable for non-conveyance.

### Source of call

Data was recorded on the source of request for the APP Team for 732 cases; 44.4% of calls were primary taskings to low acuity cases from NEOC, 43.2% were taskings based on a Crew Request and 11.3% of cases were ALS requests from NEOC. Of the Crew Requests, 67.4% of patients were suitable for non-conveyance in comparison to 58.2% of NEOC requests.

## Discussion

In this retrospective observational study describing the development of the Alternative Pre-Hospital Pathway service, our data demonstrates that in a cohort of patients with low acuity complaints for whom an emergency ambulance was called, 68% of them were suitable for management in the community, avoiding the need for conveyance to an emergency department. In a subpopulation of patients aged over 75, who would be considered more frail with multiple comorbidities and at a higher risk of requiring hospital admission, [18, 19] 62% of these patients were suitable for non-conveyance.

### Benefits

An emergency department is not a benign environment; noise, stimulation and crowding can contribute to delirium and spread of infection [20]. Providing definitive care to patients at the first point of contact in the community or referring them to the most appropriate community or specialist service is a key aim of Irish Health Service delivery as described in the Sláintecare report [10].

For the National Ambulance Service, intervening at the very start of a patient journey increases efficiency by avoiding the dispatch of an emergency ambulance, the transport of these patients to and potentially back from hospital, and the time associated with ambulance offload delays seen in many Irish EDs [21].

From a staffing perspective, in our experience, the SpRs enjoy the opportunity getting out into the community as a welcome change from the high intensity workload in the ED. Seeing patients in their home environment, getting to know the community they work in and building contacts with community services, are vital skills transferred to the clinicians’ day to day work in the ED.

### Non-conveyance initiatives

Internationally non-conveyance rates vary greatly. Rates in the UK are 23–51%, [13] 20.4% in the Netherlands, [22] and between 10 and 22% in Sweden [12]. With a 68% non-conveyance rate, our data demonstrates that an APP Team is an effective model to reduce conveyances to hospital. This is consistent with the 67% non-conveyance rate reported by London’s Air Ambulance Physician Response Unit [23]. This patient-centred care assists in releasing emergency ambulances back into the system and relieving pressure on Emergency Departments.

Outside of Community Emergency Medicine, a number of other initiatives have been developed to facilitate non-conveyance of 112/999 callers. In the UK, Falls Response Teams have been developed to attend to and assess elderly patients who have fallen [24]. In Ireland, a number of hospital outreach projects targeting frail elderly patients have commenced including the Pathfinder Alternative Care Pathway (Pathfinder ACP) [25] and Wicklow Frailty Response Team [26].

### Team skill-mix

In this dataset, 8.5% of the calls attended to by the APP Team involved the provision of Advanced Life Support (ALS) or Critical Care by the APP Team. The focus of the APP team is on targeting low acuity calls, however the combination of an Emergency Medicine Doctor and EMT skill-mix makes the team a very flexible and useful resource for the Ambulance service with an ability to cover a broad spectrum of presentations from discharging low acuity, to opportunistically providing ALS and Critical Care as required. Our staffing is very similar to other Community Emergency Medicine initiatives in the UK most of which consist of a doctor and an EMS Clinician [27]. This contrasts to non-conveyance initiatives in Europe which are led by Specialist Ambulance Nurses [28] and community paramedics in North America [29].

A suitably qualified doctor is an integral component of a low acuity alternative pathway service. They can provide definitive care and discharge patients on scene, something which is not yet afforded to paramedic or advanced paramedic practitioners in their scope of practice in Ireland. Doctors can treat a wide variety of low acuity presentations including frail elderly, minor injuries and patients with addiction and social issues. In addition, familiarity with in-house specialities and community services enables real-time engagement with appropriate services as opportunities arise to effect bespoke non-conveyance pathways.

### Tasking

Identifying appropriate 112/999 calls where the APP Team can add benefit to patient care is challenging and is something that many specialist pre-hospital services struggle to achieve [30]. AMPDS is a triage tool used for emergency calls. It is designed to identify patients with time critical illness and injury facilitating prompt dispatch of emergency resources [31]. It was not developed to identify patients suitable for non-conveyance [32]. Acuity and complexity of illness are independent. Patients can be identified as low acuity by AMPDS but may have extremely complex care needs. Only 58% of primary taskings from NEOC were suitable for non-conveyance.

### Generalisability

Through engaging appropriate stakeholders (Table 2), an APP Team service is easily replicable in other jurisdictions and offers the opportunity to avoid, where appropriate, conveyance to the ED. It is also a platform for integrating specialist hospital and community services, in certain cases aided by telemedicine, thereby enhancing the package of care delivery. This concept is similar to community paramedicine and mobile integrated care models used throughout North America [29] and guideline directed non-conveyance by specialist ambulance nurses in Europe [12].

### Limitations

This study was performed in a mixed urban and rural environment which should be taken into consideration when adopting elsewhere. The efficiency and utilisation may not be realised in a rural area due a lower volume of patients and increased travel times. Conversely a rural environment has the opportunity to save more emergency ambulance travel time, ensuring sparse resources are available for seriously ill and injured patients. As a retrospective observational study, our data is reliant on accurate documentation of the case by the treating clinician. A large proportion of the study period included the COVID-19 pandemic with varying levels of public health measures which may have impacted the study; other jurisdictions have demonstrated an impact on ambulance service call volumes [33] and emergency department attendances [17].

Patient and staff feedback did not constitute a part of this study but is something that should be reviewed to further document the impact of the service and the motivations and challenges faced by the clinicians [34].

## Conclusion

Increased demand for emergency care and an aging population is necessitating a re-design of traditional models of emergency care delivery. We describe the Alternative Pre-Hospital Pathway Service, delivered by an EMT and an Emergency Medicine SpR responding to low acuity calls. This service achieved a 68% non-conveyance rate; our data demonstrates that a community emergency medicine outreach team in collaboration with the National Ambulance Service offering Alternative Pre-Hospital Pathways is an effective model for reducing conveyances to hospital.

## Supplementary Information

**Additional file 1:.** Categorisation of Patient Presentations. Description of the patient presentations associated with each call category.

## Data Availability

De-identified case data available from corresponding author (https://orcid.org/0000-0002-2128-1759) upon reasonable request.

## Abbreviations

ALS: Advanced Life Support
AMPDS: Advanced Medical Priority Dispatch (AMPDS)
AP: Advanced Paramedic
APP: Alternative Pre-Hospital Pathway
ATC: Assessment and Treatment Centre
CEM: Community Emergency Medicine
CPGs: Clinical Practice Guidelines
CREC: Clinical Research Ethics Committee
CUH: Cork University Hospital
ED: Emergency Department
EM: Emergency Medicine
EMS: Emergency Medical Service
EMT: Emergency Medical Technician
ICV: Intermediate Care Vehicle
iPMS: Integrated Patient Management System
IQR: Interquartile Range
MMS: Mobile Medical Service
MUH: Mercy University Hospital
NAS: The National Ambulance Service
NEOC: National Emergency Operations Centre
PCR: Patient Care Report
PHECC: The Pre-Hospital Emergency Care Council
RTC: Road Traffic Collision
SpR: Specialist Registrar
STROBE: Strengthening the Reporting of Observational Studies in Epidemiology
UCC: Urgent Care Centre
