# Supplementary material for: The Alternative Pre-hospital Pathway team: reducing conveyances to the emergency department through patient centered Community Emergency Medicine
Source: BMC Emerg Med. 2021 Nov 18;21:138. doi: 10.1186/s12873-021-00536-x (PMC8601091; doi:10.1186/s12873-021-00536-x)
Supplement: Supplementary file 1 — Additional file 1:. Categorisation of Patient Presentations. Description of the patient presentations associated with each call category. [file 12873_2021_536_MOESM1_ESM.pdf]

Additional File 1

Categorisation of Patient Presentations

Seizure	Patients reporting seizure activity.
Fall	Falls (excluding falls from significant height)
Medical	diabetic emergencies, confusion, lethargy, general decline, headaches, feeling generally unwell, dizziness, decreased mobility, poor oral intake and delirium.
Syncope	Including pre-syncopal symptoms
RTC	Patients involved in Road Traffic Collisions
Gastro	GI Bleeding, Vomiting & Diarrhoea
Drug / Alcohol Related	Alcohol intoxication, recreational self-poisoning and accidental ingestions
Burns / Fire	including Smoke inhalation
Respiratory	Exacerbations of COPD or Asthma, Lower Respiratory Tract infections excluding COVID-19
Mental Health	Anxiety, Depression, Psychosis and other mental health disorders
MSK	Musculoskeletal Disorders (excluding acute injuries) e.g. back pain, knee pain
ENT	Epistaxis, tonsillitis, sore throat and suspected upper respiratory tract infections
Chest Pain / Palpitations	Atraumatic chest pain or palpitations.
MISC	Peg Tube replacement, ring removal request, trapped patient
Urology	catheter problems, urinary tract infections, renal colic and haematuria
Wounds	Ulcers, pressure sores, bleeding varicose veins
Surgical	Abdominal Pain, Vascular Presentations, Orthopaedic Issues (excluding acute injuries)
Injuries: Wounds	Wounds sustained from acute trauma.
Cardiac Arrest	Cardiac Arrests including where resuscitation was not attempted.
Palliative	Patients known to palliative care team at end stages of life
Social	Medically fit patients suffering homelessness, Elderly home help issues, poor social circumstances
Allergy	Allergic Reactions
Obs & Gynae	PV Bleeding, Pregnancy related illnesses
Choking	Patients choking on food or other foreign body.
Injuries: Fractures	Acute fractures from trauma.
Injuries: Head Injury	Head Injuries from trauma.
Injuries: Other	Soft tissue injuries
Injuries: Major Trauma	Assault, Fall from height (e.g. ladder/roof)
Neurological	New Focal neurological deficit, Ataxic.
Oncology	Symptoms related to known malignancy or treatment
COVID-19: Related	COVID-19 Positive, COVID-19 Suspected, COVID-19 Swabs
